# Better than expected performance effect depends on the spatial location of visual stimulus

**DOI:** 10.1038/s41598-024-82146-8

**Published:** 2025-01-02

**Authors:** Soodeh Majidpour, Mehdi Sanayei, Reza Ebrahimpour, Sajjad Zabbah

**Affiliations:** 1 School of Psychology, Allameh Tabataba’i University, Tehran, Iran; 2 School of Cognitive Sciences, Institute for Research in Fundamental Sciences (IPM), Tehran, Iran; 3 Center for Cognitive Science, Institute for Convergence Science and Technology (ICST), Sharif University of Technology, Tehran, Iran

**Keywords:** Perception, Decision-making, Spatio-temporal integration, Neuroscience, Psychology

## Abstract

The process of perceptual decision-making in the real world involves the aggregation of pieces of evidence into a final choice. Visual evidence is usually presented in different pieces, distributed across time and space. We wondered whether adding variation in the location of the received information would lead to differences in how subjects integrated visual information. Seven participants viewed two pulses of random dot motion stimulus, separated by time gaps and presented at different locations within the visual field. Our findings suggest that subjects accumulate discontinuous information (over space or time) differently than when it is presented continuously, in the same location or with no gaps between them. These findings indicate that the discontinuity of evidence impacts the process of evidence integration in a manner more nuanced than that presumed by the theory positing perfect integration of evidence.

## Introduction

We make decisions all the time, from selecting our future career to the brand of our clothing. These decisions require gathering evidence for different options and combining it to make a final choice. This information may come at various times and from multiple sources. For instance, when approaching an intersection while driving, you rely on data about the positions of other vehicles, potential obstacles like potholes, and traffic signals. Much of this information is obtained through peripheral vision. But how do we integrate all the information reaching us from different locations and separated by temporal gaps?

Various efforts have been made to discover the neural^1–4^and behavioral^5–7^representations of the decision-making process. Studies show that integration of sequentially sampled evidence over time until reaching a threshold (i.e., accumulation-to-bound model) can explain these representations during a decision-making task^5,8–16^. This process of integration and its termination, is also found to be correlated with the ramping to a stereotype activity in several areas of the brain including the cortical and subcortical structures^2,3,17–19^.

While the classic version of the accumulation-to-bound model assumes a time-independent accumulation process^5,20–22^, a number of models include some form of time-dependent accumulation^15,23–26^to account for information leakage or attention modulation. In our daily experiences, we gather the information required for decision-making, which is often presented as distinct pieces of evidence^27–29^, and not as a continuous stream at a single location. Several preceding studies have tried to integrate elements of real-life contexts inherent in perceptual decision-making into the conventional paradigms of visual tasks^6,30–33^. Previous studies on the spatial integration of visual stimuli have focused on feature fusion^34^, spatial statistics^35^, and face perception^36–38^.

To explore how the passage of time influences the accumulation of visual evidence, researchers employed temporally discrete paradigms of established perceptual decision-making tasks by introducing temporal gaps between successive pulses of stimuli^39–41^. The results have shown that the accuracy of the ultimate decision is not affected by the duration of the gap between discrete pieces of evidence, and participants integrate them with no loss (i.e., perfect integration). However, the observers extracted more information from the last pulse compared to the first one (sequence dependence or recency effect). It has also been shown that predictions of perfect accumulation models underestimated participants in double-pulse trials (i.e., better-than-expected-accuracy). It is important to note that these behavioral effects did not align with the predictions made by the proposed attractor-based decision-making models^39,42^. This discrepancy highlights the significance of these findings in enhancing our understanding of the mechanisms underlying decision-making.

The recency effect may help explain why participants achieve better performance than the expected accuracy, calculated from their accuracy in the single-pulse trials. This suggests that participants might gather more evidence from the second pulse. Researchers have identified several reasons for the recency effect. One explanation involves a time-dependent attention mechanism^40^, which indicates that participants pay more attention to the most recent information. Additionally, the concept of adaptive gain in information processing^43^ suggests that the ability to process evidence is quickly adjusted, leading to a recency bias.

As mentioned earlier, several studies have examined how the temporal dimension affects the integration of evidence. However, to our knowledge, there is a gap in research regarding the role of the spatial dimension and its interaction with time in the evidence integration and decision-making process. In other words, the effects so far have occurred when pulses of evidence were presented in the same spatial position. In the present study, we aimed to investigate whether the similarity of spatial position may manipulate these effects. We used Random Dot Motion (RDM) which is a well-established stimulus in perceptual decision-making research. In this setup, participants observe a field of dots that a subset of them move in one direction while others move randomly. This allows researchers to investigate the neural mechanisms underlying decision-making^44,45^.

In the present study, two pulses of RDM stimuli were presented in either the same or different locations within the para-foveal vision of the participants. While participants focused on the center of the screen, two stimuli were shown with or without a time gap between them, either above or below the fixation point, depending on the specific location condition. By fitting logistic regression models, we tested whether spatial and temporal separation of evidence had any influence on the eventual decision and on the previously observed effects. We found that when RDM pulses were presented at different locations, subjects could not perfectly integrate the entire evidence provided by each of the pulses. Moreover, we showed that the previously observed effects were spatially dependent and some of them vanished when the pulses of discrete evidence were presented at different locations.

## Results

**Fig. 1:**
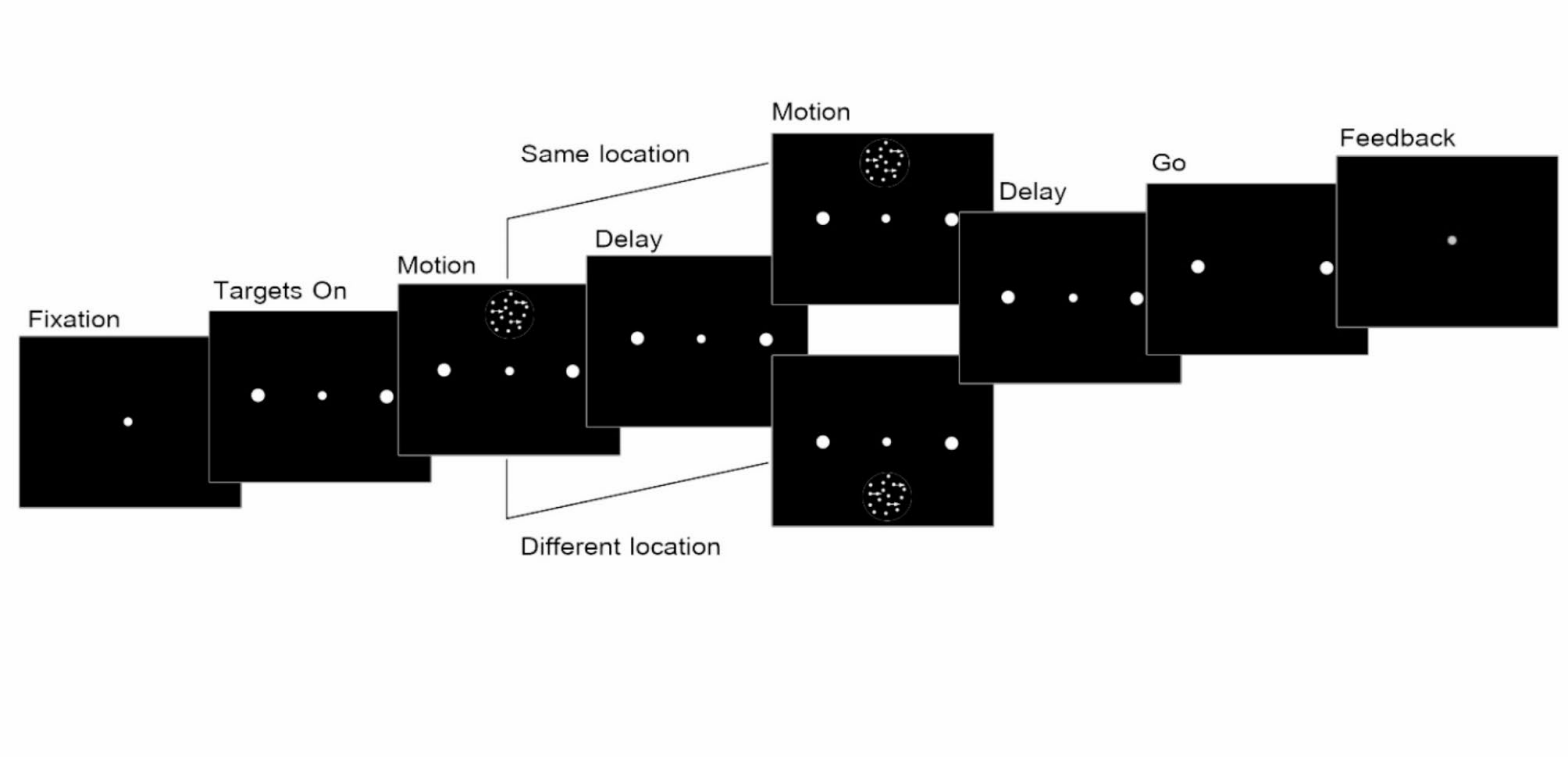
Spatio-temporally discrete version of direction discrimination task. Subjects fixated on a central fixation point and whenever they were ready, they pressed the Space key and targets appeared on both sides of the screen. After an unpredictable delay, a pulse of motion was presented on center, top, or bottom of the screen. In the single pulse trials, following a random delay, ‘Go’ signal appeared and in double pulse trials, after the first pulse and a delay of 0, 480 or 1080ms, the second pulse of motion appeared on either the same or different location relative to the first pulse. Subjects reported direction of dots and visual feedback was provided (green indicated correct and red showed wrong answers).

Seven human participants completed several blocks of single or double-pulse trials presented in either periphery (top or bottom) or the center of their vision. The two peripheral pulses in each trial were presented at either the same (SL) or different locations on the screen (DL). The presented stimulus in each pulse was a set of random dots moving to the right or left for 150ms. At the end of each trial, subjects reported their perceived direction by pressing the right or left arrow key (Fig. 1). In order to avoid uncertainty about the location of stimuli, the sequence of presentation was fixed within each session, and participants were informed of it in advance. We collected 11,836 trials in the center, 8940 in top-top, 9151 in bottom-bottom, and 8956 in top-bottom, and 9064 trials in bottom-top conditions, resulting in a total of 47,947 trials.

### Participants improved their decision by using information within each pulse

**Fig. 2:**
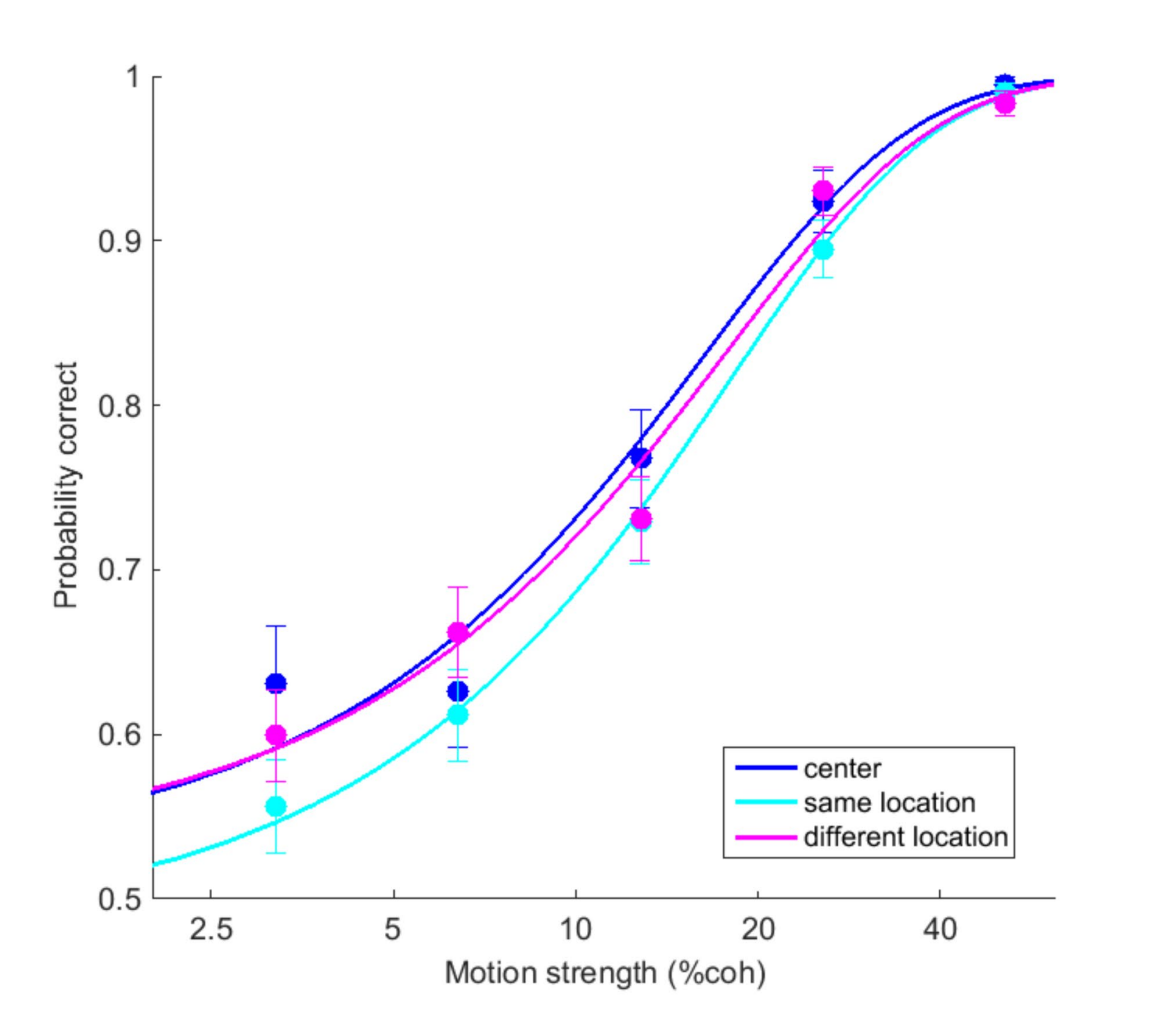
Performance (circles) and fitted psychometric functions (lines) of single pulse trials separated by location conditions (blue: center, cyan: same and magenta: different). Error bars indicate SEM estimated from binomial distribution.

**Fig. 3:**
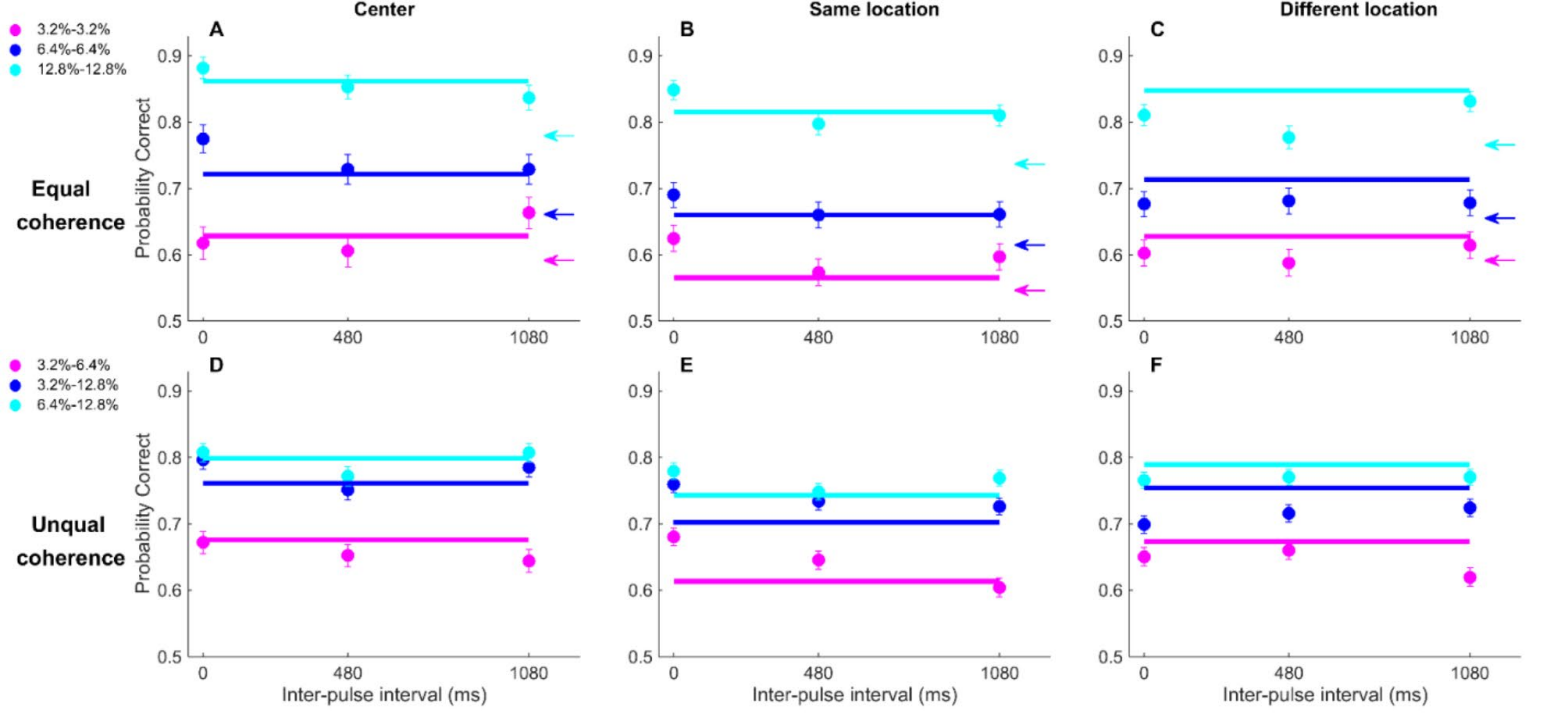
Performance of subjects in double pulse trials of the three location conditions. Arrows in A-C indicate the performance in single pulse trial, circles represent the performance of subjects in double pulse trials and solid lines show the estimated performance of a perfect integrator. Subjects performed at the level of (**A**, **D**, center), better than (**B**, **E**, same), or worse than (**C**, **F**, different) the perfect integrator. Error bars indicate SEM estimated from binomial distribution.

First, we looked at the performance of participants in the single-pulse trials as a function of coherence conditioned on the RDM location (Fig. 2). Despite the brief presentation of the stimulus (150ms), subjects showed a graded level of performance depending on the stimulus strength and reached close to 100% performance for the coherence of 51.2%. This pattern was very similar between conditions. While participants showed a bias to the right at the center location (Blue circles and line, Eq. 2, β_0_ = 0.20 ± 0.08, *p* = 0.01), there was no significant bias observed in other conditions (Eq. 2, SL: cyan, β_0_ = −0.12 ± 0.06, *p* = 0.07; DL: magenta, β_0_ = −0.08 ± 0.07, *p* = 0.24). Performance in single-pulse trials in the DL (73.76%) was higher than in the SL (72.02%), but this difference did not reach a significance level (repeated-measures ANOVA, F = 2.10, df = 2, *p* = 0.16). Next, we looked at the performance in double-pulse trials. When both pulses had the same coherence, in center (Fig. 3A), same (Fig. 3B), and different (Fig. 3C) locations, performance in the double-pulse trials (circles) was better than the corresponding performance in the single pulse trials (colored arrows).

## The effect of gap duration varied based on the location of the stimuli

Looking at the results of the Eq. 4, we observed a significant difference between the center and DL (Eq. 4, *p* < 0.01), while there was no significant difference in the center vs. same, or same vs. different comparisons (Eq. 4, p’s > 0.05). At the SL condition, we found that the gap between the two pulses had a significant effect on performance (Fig. 3B & E, Eq. 3: β_3_ = −0.24 ± 0.11, *p* = 0.03). This effect was not observed for the central condition (Fig. 3A & D, β_3_ = 0.1 ± 0.14, *p* = 0.47), or DL conditions (Fig. 3C & F, β_3_ = −0.13 ± 0.11, *p* = 0.24). In other words, while gap ($\:{{\upbeta\:}}_{3}$ in Eq. 3) had a significant effect on performance in the same condition (i.e., top-top and bottom-bottom), it did not reach a significance level in the center and different conditions.

## Dissimilarity in the location of pulses reversed the better-than-expected effect

In order to test the hypothesis of location dependency of the better-than-expected effect, we compared the performance of participants with the expected performance computed by a perfect integrator in different conditions of the experiment. The perfect integrator estimates the performance of double pulses based on the performance of the single pulses, assuming perfect integration of evidence (Eq. 6). Our results suggest that while subjects achieved performance at the level of the perfect integrator in the center condition (Fig. 3A, Eq. 5: β = 0.03 ± 0.04, *p* = 0.80), they outperformed the expected accuracy at the SL condition (Fig. 3B, Eq. 5: β = 0.07 ± 0.03, *p* = 0.008), and underperformed in the DL condition (Fig. 3C, Eq. 5: β = −0.17 ± 0.03, *p* < 0.001). This pattern was the same when we re-ran the analysis for double pulse trials with unequal coherences (Fig. 3D-F, Eq. 5: center: β = −0.01 ± 0.03, *p* = 0.35, SL: β = 0.14 ± 0.02, *p* < 0.001, DL: β = −0.16 ± 0.02, *p* < 0.001).

## Sequence dependency effect was location dependent

**Fig. 4:**
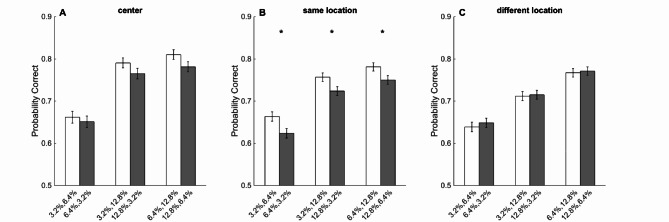
The effect of pulse sequence on performance. **A**, **B**) Accuracy in weak-strong (light shade) trials were higher than the strong-weak (dark shade) trials in the central and same location conditions, respectively. **C**) Both pulses contribute to the same degree to the performance when pulses are presented in different locations of the visual field. Error bars indicate SEM estimated from binomial distribution.

As shown in previous work of Kiani et al. and Tohidi-Moghaddam et al., better-than-expected performance is the result of the sequence dependency effect (i.e., extracting more information from the second pulse compared to the first pulse). As mentioned above, our results also showed this effect in the SL condition (i.e., top-top and bottom-bottom) but not in the DL condition (i.e., top-bottom and bottom-top, Fig. 4). To test if sequence dependency of performance is also observed here, we divided the double-pulse trials based on the sequence of weak and strong coherences (e.g., 3.2–6.4% vs. 6.4–3.2%, Fig. 4). In the SL condition, we found that performance was significantly higher when the second pulse was stronger (Fig. 4B, 3.2–6.4 = 66.33%, 6.4–3.2 = 62.36%, *p* = 0.013; 3.2–12.8 = 75.65%, 12.8–3.2 = 72.36%, *p* = 0.024; 6.4–12.8 = 78.12%, 12.8–6.4 = 75.01%, *p* = 0.028, Wilcoxon Rank Sum). We observed the same pattern of results for the central location (Fig. 4A, 3.2–6.4 = 66.16%, 6.4–3.2 = 65.09%; 3.2–12.8 = 79.05%, 12.8–3.2 = 76.53%; 6.4–12.8 = 81.01%, 12.8–6.4 = 78.13%), though the difference did not reach a significance level (p’s > 0.05). For the DL condition, we observed that the first pulse had a stronger effect on performance (Fig. 4C, 3.2–6.4 = 63.86%, 6.4–3.2 = 64.82%; 3.2–12.8 = 71.18%, 12.8–3.2 = 71.48%; 6.4–12.8 = 76.71%, 12.8–6.4 = 77.10%), but these differences did not reach a significance level either (p’s > 0.05).

**Fig. 5:**
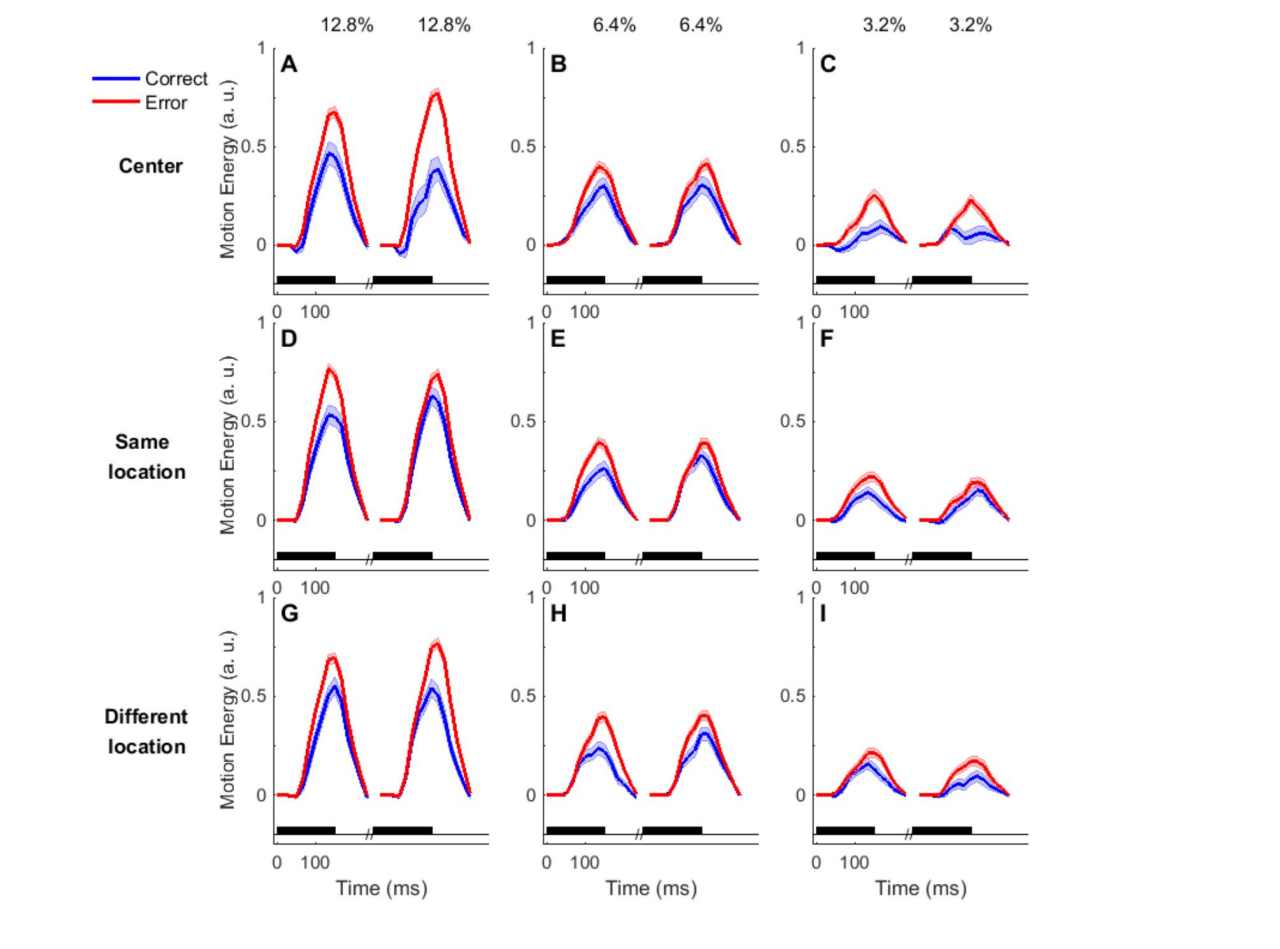
Motion Energy of double pulse trials with same coherence and nonzero gaps. Motion energy of correct (red) and error (blue) trials at the center (**A**-**C**), same (**D**-**F**) and different location (**G**-**I**). The black horizontal boxes indicate the period of presentation of each pulse. The units of motion energy are arbitrary and shaded area around the mean indicates SEM.

Due to the stochasticity in the RDM stimulus, an analysis of motion energy for each pulse can give us a better understanding of the effect of the pulse sequence on performance. This analysis was done on a portion of 30,058 trials of the data with gap durations greater than 0ms. Results from motion energy analysis of the data of central location confirmed the stronger influence of the second pulse on the accuracy of subjects (Fig. 5, top row, Eq. 13: β_3_ = −0.07, *p* = 0.04; Eq. 14, β_4_ = 0.17, *p* = 0.001). However, it seems that in the SL and DL conditions, both pulses contribute almost equally to the performance (same: Eq. 13: β_3_ = 0.004, *p* = 0.88; Eq. 14: β_4_ = −0.01, *p* = 0.75; different: Eq. 13: β_3_ = −0.02, *p* = 0.44; Eq. 14: β_4_ = 0.03, *p* = 0.43).

## Discussion

Making a decision in the real world usually requires collecting information from various sources that are often presented in different parts of our visual field. In this study, with the goal of getting closer to real-world conditions, we examined both time and space dimensions simultaneously. Participants were asked to discriminate the direction of motion of two pulses of RDM displayed in the center or periphery of their visual field, and at the same or different locations on the screen. We observed that performance in the central vision was generally better than in peripheral conditions, which is not unexpected as previous work has shown that spatial resolution decreases with eccentricity^46–49^. More interestingly, there were differences in the process of evidence accumulation across different conditions such that participants performed better than expected (from a perfect integrator) when both pieces of information were presented at the same location in periphery and worse than expected when presented at different locations. The sequential effect of the second pulse on the performance also depended on the location of the presentation of the stimuli. Previous studies that showed subjects integrate information at a perfect level, or better than the perfect integrator, all presented stimuli at the center^39–41,50^. As far as we know, our study is the first to systematically investigate the effects of the location of evidence on subjects’ performance.

Considering the temporal dimension and in line with previous works^39,40^, we observed that the gap duration did not show a significant effect on the performance of the subjects in the center condition. We observed the same pattern of results for the DL condition. However, for the SL condition, the gap between pulses had a significant effect on subjects’ performance (p-value = 0.03). Previous work that studied decision-making with discrete stimuli^39–41,50^ showed that when both stimuli were presented at the center of vision, the second piece of information had a stronger effect on choice than the first one. We replicated those findings at the center of vision and extended them to the same peripheral location. When we presented our stimuli at different locations, we found that the stronger effect of the second pulse was no longer present.

The perfect integrator model is calculated based on the performance of the single pulse trials in each condition. The single-pulse figure is slightly higher for the DL condition in comparison to the same peripheral condition (Fig. 2). Even though this improvement did not reach the significance level, the resulting values for the perfect integrator of the different-location condition seem to be larger than the ones for the same-location condition. Therefore, the worse-than-perfect integration in the DL condition could be in part due to the higher performance in the single-pulse trials. On the other hand, the DL condition appears to necessitate shifting spatial attention between the two pulses, a process taking approximately 150ms^51^. This becomes particularly pronounced at the 0ms gap, where participants have no time between successive stimuli, potentially causing them to miss information from the latter part of the first pulse and the initial part of the second pulse.

Research on attentional allocation across spatial discontinuities suggests that such shifts of attention can reduce the precision of perceptual integration, especially when attention must be quickly reallocated between locations. Consistent with the spotlight theories of attention, shifts in spatial attention can reduce the resources available for processing stimuli that are spatially discontinuous^52–54^. Moreover, covert attention shifts are accompanied by efforts to suppress saccadic eye movements toward the stimulus location, increasing cognitive load^55,56^. Under high perceptual and cognitive load, attentional resources are fully dedicated to goal-relevant stimuli, leaving limited capacity to integrate information across spatially separated locations. This pattern helps explain why attentional shifts and resource limits under high perceptual load can impact integration performance for stimuli presented at different locations^57,58^.

**Fig. 6:**
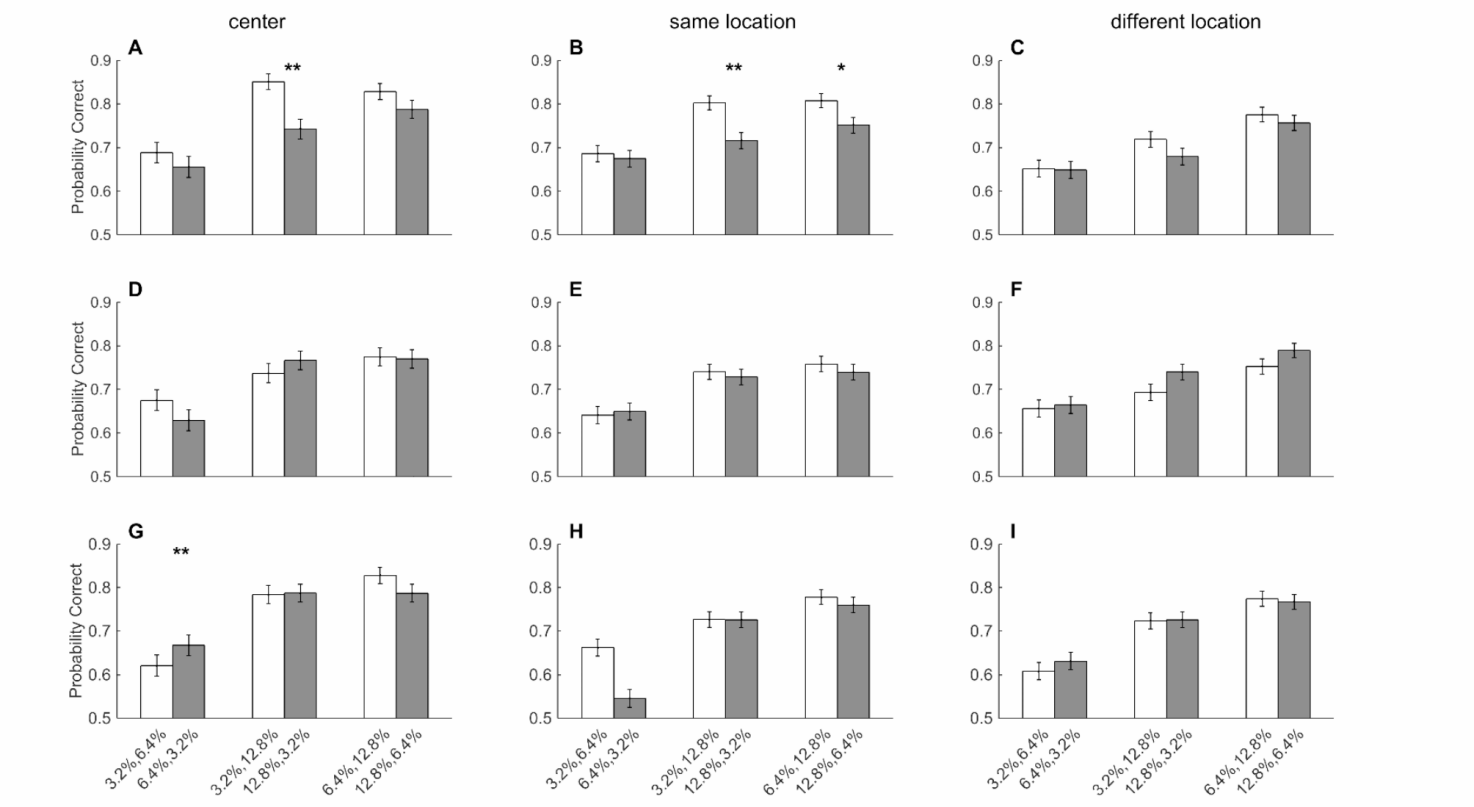
Performance in weak-strong (light shade) vs. strong-weak (dark shade) trials, separated based on the gap duration. **A**-**C**) 0ms, **D**-**F**) 480ms, **G**-**I**) 1080ms. The effect of stronger second pulse is evident in trials with zero inter-pulse interval.

Comparison of the accuracy in weak-strong and strong-weak trials showed a pattern of the stronger effect of the second pulse in the same-location conditions, both in the fovea and the periphery, but the effect was significant only in the peripheral condition (Fig. 4). On the other hand, the motion energy analysis showed the sequential effect to be significant merely in the central condition (Fig. 5). Considering the exclusion of the gap of 0ms in the motion energy analysis, we decided to separate the plots presented in Fig. 4 based on the gap duration between the two pulses (Fig. 6). Interestingly, we observed that the privileged effect of the second pulse at the center and SL conditions was limited to the 0ms gap, and as the gap duration increased, the significant effect of the second pulse on subjects’ performance decreased. These results suggest the role of continuity in the stream of evidence on the perception of the second piece of information. When we break this continuity in time or space, both pulses contribute to the same degree to the final decision. Previous studies on stimulus discontinuity have shown that spatial separation can impair both category learning^59^and temporal perception^60^, highlighting spatial attention’s critical role in visual perception.

The effect of the second pulse observed in the 0ms gap duration of the central and SL conditions could be due to some manifestation of recency or confirmation bias^43,61–64^. The higher performance in the 0ms gap, compared to other gaps, shows that subjects gathered more information from the second pulse. One reason could be that viewing the first pulse increases sensitivity to the observed data either in the low-level visual circuits or through evidence integration in higher levels of the decision-making system. This increase in sensitivity could be what decays during the gap duration, leading to the absence of the sequential effect and lower performance in the gap durations longer than 0ms. Additionally, Introducing a break in the process of data accumulation could make the observer treat the pulses as distinct pieces of information, leading to a categorical judgment of the first pulse, which lessens sensitivity to the newer information^34,65,66^. Previous studies have also shown the effect of attention and top-down procedures leading to a flexibility during perceptual decision-making in a changing environment^67–70^.

More research is needed to see how the brain integrates discrete pieces of information over space and time with a wider set of stimuli and conditions. Specifically, our study did not encompass different-location conditions involving pulses from the center and the periphery in order to mitigate the distinct effects attributed to each visual area. While incorporating more complex scenarios would enhance our comprehension, the limitation on the number of conditions compelled us to focus on the most informative scenarios. The significance of peripheral vision in daily navigation and directing attention towards important or potentially threatening events is well-established^71,72^. In our study, stimuli were presented at an eccentricity of 5 visual degrees from the fovea. Considering previous research indicating a decline in visual feature acuity with increases in eccentricity^47,49,51^, it is reasonable to explore the impact of larger eccentricities in a setting similar to ours. We also believe that utilizing neuro-imaging techniques such as EEG and fMRI with discrete stimuli could lead to a better understanding of the underlying mechanisms and help clarify the role of attention in the accumulation of this type of incoming information.

To make a decision in real life, it is not common to receive pieces of information from the same location. In most natural conditions, given the movement of objects, head and body, we need to accumulate information reaching us from different places. Our results indicate that individuals incorporate discontinuous information in a distinct manner compared to its continuous form and thus, the discrete nature of evidence affects the integration process of such information.

### Methods

#### Participants

Nine human subjects (5 female, aged 23–43 years), with normal or corrected-to-normal vision, were included in this study. The participants were chosen by convenience sampling and all of them filled out an informed consent form before their attendance. Subjects underwent a rigorous training procedure before the main experiment to reach a predefined level of accuracy and reaction time (see below). Two subjects (both female) failed to reach the required minimum performance in the first training phase and were excluded from the study; thus, all the reported results are derived from a set of seven participants. This research received approval from the Ethics Committee of Allameh Tabataba’i University (ID: IR.ATU.REC.1401.022) and was conducted in accordance with the Declaration of Helsinki.

## Stimulus

In this study, we used a Random Dot Motion (RDM) paradigm in which participants were asked to decide the net motion direction of a set of dots moving left or right with coherence varying between trials (0, 3.2, 6.4, 12.8, 25.6, or 51.2%). The more coherent the dot motion, the higher the probability of providing a correct answer. Detailed information on the RDM properties is as follows: dot density of 16.7 dots/deg^2^/s, dot size of 3 × 3 pixels (0.075°), velocity of 5°/s, and aperture size of 5°. The stimulus consisted of white dots moving against a black background, located in one of three locations: the center, above (top), or below (bottom) the fixation point, at the eccentricity of 5°. In each block of 120 trials, with a fixed aperture location, each set of dots was displayed on the screen for one frame (16.7ms) whose position got updated every three frames, i.e. the position of dots in the first frame was updated in the fourth frame and so on, as previously has been done^7,73^.

### Task design

Each trial began by presenting a red fixation point (0.2° diameter) at the center of the screen. After subjects fixated on the fixation point and pressed the space bar, two white targets (0. 65° diameter) were presented on the right and left sides of the screen (10° eccentricity). After a random delay (500-800ms, truncated exponential distribution), the motion stimulus was presented at a circular aperture at the center, top or bottom location, depending on the block condition.

In each block, we had two types of stimulus presentation; ‘*single-pulse’*, in which a single pulse of motion was shown for 150ms (0–51.2%); and ‘*double-pulse’* trials, in which two pulses of motion (each 150ms) were shown successively. The coherence of each patch of dots could be 3.2%, 6.4% or 12.8%. Both pulses had the same net direction and participants knew this fact in advance. The interval between the two pulses was randomly chosen from 0, 480 and 1080ms intervals. Each block consisted of 108 double-pulse and 12 single-pulse trials. Subjects were instructed to give their answer as accurately as possible after the offset of the fixation point (go cue). Subjects received visual feedback based on the accuracy of their responses, with a green circle indicating a correct response and a red circle representing an incorrect answer.

### Task procedure

Stimuli were presented on a 24˝ LED monitor (screen resolution: 1920 × 1080, refresh rate: 60 Hz) at a distance of 60 cm in a dark room. To ensure that subjects’ gaze remained on the fixation point, we monitored the position of the left eye throughout the experiment (EyeLink 1000, SR Link, Canada). To stabilize the subject’s head, a chin rest and a forehead bar were used. The task was implemented in MATLAB using Psychtoolbox-3^74^. In the main experiment, each subject completed 1,100-2,900 trials (120 trials per block) for each condition. We had three main conditions including the center location (CL): two pulses were presented at the center; the same location (SL): both pulses were presented at top or bottom; and the different location (DL): the first pulse was presented at the top and the second one at the bottom, or vice versa.

### Training

All subjects participated in a procedure of extensive training which included two distinct phases. The first phase of training consisted of at least two sessions in which three blocks of 120 trials of the RDM stimulus were displayed for a fixed duration of one second. The process was repeated for all stimulus presentation locations (center, top and bottom) separately, until the subject reached an average performance of 85% in a session. Upon successfully finishing the phase one, subjects entered the second phase, in which the presentation of the stimulus in each trial continued until subject’s choice (i.e., free response). The maximum duration of viewing the stimulus on each trial was set to five seconds. By reaching an average performance of 85% in a session and in all the conditions, a subject would be ready to start the main experiment. Before starting the main task, all subjects passed several training sessions (on average, each subject performed 19 blocks to be trained in all conditions) to attain high performance levels (i.e., 85%).

### Data analysis

Having the binary nature of the outcomes (either true or false), in order to assess the impact of different parameters of the stimulus in this experiment, we used multiple logistic regression models. Logit[P] stands for log$\:\left(\frac{p}{1-p}\right)$ and β_i_ implies fitted coefficients. The fitting method was maximum likelihood under a binomial error model (i.e., a GLM).

The probability of a correct choice for single pulse trials was estimated as:

1 $$\:\text{Logit}\left[{P}_{correct}\right]={{\upbeta\:}}_{0}+{{\upbeta\:}}_{1}\text{C}$$

where C was the motion coherence.

A modified version of Eq. 1 was applied to check for right/left bias:

2 $$\:\text{Logit}\left[{\text{P}}_{\text{r}\text{i}\text{g}\text{h}\text{t}}\right]={{\upbeta\:}}_{0}+{{\upbeta\:}}_{1}{\text{C}}_{\pm\:}$$

Where C_+_ corresponds to rightward motion and C_−_ to leftward motion.

To find the impact of time gap between the two pulses in double-pulse trials we used:

3 $$\:\text{Logit}\left[{\text{P}}_{\text{c}\text{o}\text{r}\text{r}\text{e}\text{c}\text{t}}\right]={{\upbeta\:}}_{0}+{{\upbeta\:}}_{1}{\text{C}}_{1}+{{\upbeta\:}}_{2}{\text{C}}_{2}+{{{\upbeta\:}}_{3}\text{T}+{\upbeta\:}}_{4}{\text{C}}_{1}\text{T}+{{\upbeta\:}}_{5}{\text{C}}_{2}\text{T}$$

where T indicates the gap duration, and C_1_ and C_2_ indicate coherence in the first and second pulse, respectively.

To see whether location of the dots patch had any contribution to the probability of giving a correct answer, we defined another equation, where L indicates the location of the dots patch including three categories of ‘center’ (L = 0), ‘same’ (L = 1) and ‘different’ (L = −1):

4 $$\:\text{Logit}\left[{\text{P}}_{\text{c}\text{o}\text{r}\text{r}\text{e}\text{c}\text{t}}\right]={{\upbeta\:}}_{0}+{{\upbeta\:}}_{1}{\text{C}}_{1}+{{\upbeta\:}}_{2}{\text{C}}_{2}+{{{\upbeta\:}}_{3}\text{T}+{\upbeta\:}}_{4}{\text{C}}_{1}\text{T}+{{\upbeta\:}}_{5}{\text{C}}_{2}\text{T}+{{\upbeta\:}}_{6}\text{L}+{{\upbeta\:}}_{7}\text{L}\text{T}$$

In the next step, we fitted a logistic regression model to compare the expected accuracy of a perfect integrator with the observed accuracy:

5 $$\:\text{Logit}\left[{\text{P}}_{\text{c}\text{o}\text{r}\text{r}\text{e}\text{c}\text{t}}\right]=\text{Logit}\left[{\text{P}}_{\text{e}}\right]+{\upbeta\:}$$

where P_e_ is the expected probability derived from a perfect integrator. If β ends up with a positive value, then observed accuracy is higher than expected. P_e_ was calculated as:

6 $$\:{\text{P}}_{\text{e}}=1-\phi\:(0,{e}_{1}+{e}_{2},\sqrt{2})$$

where $\:\phi\:$ is the normal cumulative distribution function, calculated as:

7 $$\:\phi\:\left(s,\mu\:,\sigma\:\right)=\:{\int\:}_{-\infty\:}^{s}N\left(v,\:\mu\:,\sigma\:\right)dv$$

where $\:N\left(v,\:\mu\:,\sigma\:\right)$ is the normal probability density function with mean of (µ) and standard deviation of (σ).

Also, e_1_ and e_2_ are pieces of evidence from the two pulses. The distribution of the evidence is calculated from the probability of correct answers from single-pulse trials:

8 $$\:{e}_{i}={\phi\:}^{-1}\left({P}_{i},0,\:1\right)\:,\:i=1,\:2$$

where P_i_ is the probability of correct response of single-pulse trials based on Eq. 1, and $\:{\phi\:}^{-1}$ is the inverse of the normal cumulative distribution function from Eq. 7.

To see the effect of pulse sequences on the probability of correct responses we used:

9 $$\:\text{Logit}\:\left[{P}_{correct}\right]={{\upbeta\:}}_{0}+{\:{\upbeta\:}}_{1}\left[{C}_{1}+{C}_{2}\right]+{\:{\upbeta\:}}_{2}\left[{C}_{2}-{C}_{1}\right]$$

where C_1_ and C_2_ are motion strength of the first and second pulse, respectively.

Furthermore, to see the interaction between two pulses (whether the stronger pulse 1 increases or decreases the effect of the second pulse), we used:

10 $$\:\text{Logit}\:\left[{\text{P}}_{\text{c}\text{o}\text{r}\text{r}\text{e}\text{c}\text{t}}\right]={{\upbeta\:}}_{0}+{\:{\upbeta\:}}_{1}{\text{C}}_{1}+{\:{\upbeta\:}}_{2}{\text{C}}_{2}+{\:{\upbeta\:}}_{3}{\text{C}}_{1}{\text{C}}_{2}$$

### Motion energy analysis

Since there are fluctuations in the RDM stimulus and the direction of dots varies in each generated frame, no two pulses carry the same amount of information regarding the direction of motion. To overcome this issue, we calculated motion energy for each pulse of every trial. Motion energy analysis is a model in visual motion perception that explains how the brain detects movement through spatio-temporal filtering. This model uses filters sensitive to specific spatial and temporal patterns, organized as pairs, to capture the direction and speed of moving objects. By combining the outputs from these filters, the brain creates a smooth and continuous measure of motion energy, allowing it to interpret real-world movement more accurately^75^.

To analyze the amount of motion energy in favor of the intended direction on each trial, we used two pairs of quadrature spatio-temporal filters^41,76,77^. Each pair was selective to either right or left. The spatial filters are defined as fourth-order Cauchy functions with both even and odd symmetry:

11 $$\:{f}_{1}\:\left(x,y\right)={{cos}}^{4}\alpha\:\:cos\left(4\alpha\:\right){e}^{-\frac{{y}^{2}}{2{\delta\:}_{g}^{2}}}\:,\:\:{f}_{2}\:\left(x,y\right)={{cos}}^{4}\alpha\:\:sin\left(4\alpha\:\right){e}^{-\frac{{y}^{2}}{2{\delta\:}_{g}^{2}}}$$

where $\:\alpha\:={\text{tan}}^{-1}\left(\frac{x}{{\delta\:}_{c}}\right)$. The filters are characterized by Gaussian-weighted sinusoids along the x-dimension, where the envelope and the period of the carrier sinusoids are determined by the fourth order and a parameter of σ_x_(0.35°). Along the orthogonal y-dimension, the filters are windowed using a Gaussian envelope parameterized by σ_γ_ (σ_γ_°). The two temporal impulse responses were derived from a linear filter characterized by the following formulation^12^:

12 $$\:{g}_{1}\left(t\right)={\left(60t\right)}^{3}\text{e}\text{x}\text{p}(-60t)\left[\frac{1}{3!}-\frac{{\left(60t\right)}^{2}}{\left(3+2\right)!}\right],\:{g}_{2}\left(t\right)={\left(60t\right)}^{5}\text{e}\text{x}\text{p}(-60t)\left[\frac{1}{5!}-\frac{{\left(60t\right)}^{2}}{\left(5+2\right)!}\right]$$

The constants in the above equations define a spatiotemporal frequency passband, which aligns with the characteristics of MT neurons.

The filters were convolved with the three-dimensional pattern of RDM stimulus and then squared and summed. The result was summated across space to give the motion energy estimate as a function of time. Finally, the net motion energy in each direction was calculated as the difference between motion energies of opponent di

13 $$\:{g}_{1}\left(t\right)={\left(60t\right)}^{3}\text{e}\text{x}\text{p}(-60t)\left[\frac{1}{3!}-\frac{{\left(60t\right)}^{2}}{\left(3+2\right)!}\right],\:{g}_{2}\left(t\right)={\left(60t\right)}^{5}\text{e}\text{x}\text{p}(-60t)\left[\frac{1}{5!}-\frac{{\left(60t\right)}^{2}}{\left(5+2\right)!}\right]$$

rections.

To test for the larger effect of the second pulse on the performance, we fit the following linear regression model:

14 $$\:\text{M}={{\upbeta\:}}_{0}+{\:{\upbeta\:}}_{1}\text{C}+{\:{\upbeta\:}}_{2}\text{E}+{\:{\upbeta\:}}_{3}\text{E}\text{S}$$

$$\:\text{E}=\left\{\begin{array}{c}0,\:\:correct\:response\\\:1,\:\:\:\:\:\:error\:response\end{array}\right.\:,\:\text{S}=\left\{\begin{array}{c}0,\:\:\:\:\:\:\:\:\:\:first\:pulse\\\:1,\:\:\:\:\:\:second\:pulse\end{array}\right.$$

Where C is the coherence of the pulse, E indicates if the response of subject was correct or wrong, S stands for the pulse sequence and M is the motion energy of that pulse. M is calculated as the summation of motion energy across a window of 200ms starting 50ms after the onset of stimulus. Since the motion energy for zero gap intervals overlapped with each other, they were omitted.

In order to compare the effect of motion energy of each of the pulses on the performance, a logistic regression model was used:

15 $$\:\text{Logit}\left[{\text{P}}_{\text{c}\text{o}\text{r}\text{r}\text{e}\text{c}\text{t}}\right]={{\upbeta\:}}_{0}+{\:{\upbeta\:}}_{1}{\text{C}}_{1}+{\:{\upbeta\:}}_{2}{\text{C}}_{2}+{\:{\upbeta\:}}_{3}({M}_{1}+{M}_{2})+{\:{\upbeta\:}}_{4}{\text{M}}_{2}$$

in which $\:{M}_{1}$and $\:{M}_{2}$ are the motion energy of the first and second pulses, respectively. For trials with equal coherence, the term $\:{\:{\upbeta\:}}_{2}{\text{C}}_{2}$ was dropped. The null hypothesis is that both pulses have the same effect ($\:{{\upbeta\:}}_{4}=0$).

## Data Availability

The datasets generated and analyzed during the current study are available in the Mendeley Data repository, https://data.mendeley.com/preview/9wk8p43z6j?a=ec44d207-4186-448c-92da-0cb2a107a64a
